# Foot Osteomyelitis Location and Rates of Primary or Secondary Major Amputations in Patients With Diabetes

**DOI:** 10.1177/10711007221088552

**Published:** 2022-05-18

**Authors:** Elin Winkler, Madlaina Schöni, Nicola Krähenbühl, Ilker Uçkay, Felix W. A. Waibel

**Affiliations:** 1 Department of Orthopedics, Balgrist University Hospital, University of Zurich, Zurich, Switzerland; 2 Infectiology, Unit for Clinical and Applied Research and Infectiology, Balgrist University Hospital, Zurich, Switzerland

**Keywords:** Diabetic foot osteomyelitis, major amputation, location of osteomyelitis

## Abstract

**Background:**

Diabetic foot osteomyelitis (DFO) often leads to amputations in the lower extremity. Data on the influence of the initial anatomical DFO localization on ultimate major amputation are limited.

**Methods:**

In this retrospective analysis, 583 amputation episodes in 344 patients (78 females, 266 males) were analyzed. All received a form of amputation in combination with antibiotic therapy. A multivariate logistic regression analysis with the primary outcome “major amputation” defined as an amputation above the ankle joint was performed. The association of risk factors including location of DFO, coronary artery disease, peripheral artery disease, neuropathy, nephropathy, and Charcot neuro-osteoarthropathy was analyzed.

**Results:**

Among 583 episodes, DFO was located in the forefoot in 512 (87.8%), in the midfoot in 43 (7.4%), and in the hindfoot in 28 episodes (4.8%). Overall, 53 of 63 (84.1%) major amputations were performed because of DFO in the setting of peripheral artery disease as primary indication. Overall, limb loss occurred in 6.1% (31/512) of forefoot, 20.9% (9/43) of midfoot, and 46.4% (13/28) of hindfoot DFO. Among these, 22 (41.5%) were performed as the primary treatment, whereas 31 (58.5%) followed previously failed minor amputations. Among this latter group of secondary major amputations, the DFO was localized to the forefoot in 23 of 583 (3.9%), the midfoot in 4 of 583 (0.7%) and the hindfoot in 4 of 583 (0.7%). In multivariate logistic regression analysis, initial hindfoot localization was a significant factor (*P* < .05), whereas peripheral artery disease, smoking, and a midfoot DFO were not found to be risk factors.

**Conclusion:**

In our retrospective series, the frequency of limb loss in DFO increased with more proximal initial foot DFO lesions, with almost half of patients losing their limbs with a hindfoot DFO.

**Level of Evidence:**

Level IV, retrospective cohort study.

## Introduction

Besides advanced ischemia, chronic osteomyelitis of the lower extremity is a frequent problem in patients with long-standing diabetes mellitus and a leading cause of lower extremity amputations.^28,50,51^ Diabetic foot osteomyelitis (DFO) is a consequence of soft tissue infection spreading to underlying osseous structures.^48^ It occurs in about 20% to 60% of diabetic foot ulcers.^10,38^ In the diabetic foot, the severity of systemic manifestations, the number of ulcers, and concomitant vascular insufficiency are influencing factors leading to major amputation, in this study defined as an amputation above the ankle joint.^23,49,60^ Only a few studies have generally linked diabetic foot lesions to the fore-, mid-, and hindfoot and analyzed their outcome in terms of healing time, recurrent lesions, and amputations.^45,56,63^ Specifically, the attribution of chronic, nontraumatic foot ulcers localized in different areas to the final clinical outcome is only reported in a few studies.^14,23,39,54^ Most of these studies included amputations in nondiabetic patients, performed for other reasons than DFO. In this retrospective cohort study, we aim to analyze the influence of the location of DFO on the probability of primary performed major amputation and the risk for conversion to secondary DFO-related major amputation. We hypothesized that in DFO, only hindfoot localization is a risk factor for both primary and secondary major amputation.

## Material and Methods

After receiving ethical approval from the local institutional review board, we performed a retrospective study of all diabetic foot patients referred to our hospital for the operative treatment of DFO from January 2000 to August 2020. All patients presented with an underlying diabetic foot ulcer. If osteomyelitis was suspected because of a positive probe-to-bone test and/or the presence of clinical symptoms, conventional radiographs (cortical erosions or destruction without prior surgery or trauma) and magnetic resonance imaging (MRI; bone marrow suppression on T1-weighted images, bone marrow edema in a fluid-sensitive MR sequence) were performed.^24,25,29^ In case of MRI contraindications, bone scintigraphy or 18F-fluorodeoxyglucose positron emission tomographic / computed tomographic scans were used instead. Intraoperative biopsy of the affected bone was further used to confirm the diagnosis and to acquire a microbiologic profile. Histology of the bone probes, however, was not mandatory and only performed in case of unclear diagnosis.

The amputations were performed in case of a clinical emergency, systemic spread of infection, or after a failed conservative therapy with antibiotics and local wound treatment. Experienced surgeons selected the amputation level based on clinical criteria; mostly on the extent of the infected bone in MRI scans, as well as the current severity of peripheral artery disease (PAD). The diagnosis of PAD was made by referral angiologists using angiologic standard methods, and the severity of PAD was based on the classification of Fontaine.^18^

For this study, we defined all amputations above the ankle joint (Syme level) as “major amputations.”^6,14,37^ All major amputations in this study were transtibial amputations. Amputations in the foot, including “internal partial foot amputations” (IPFA) defined as a simple resection of the infected bone with preservation of the surrounding healthy soft tissue and more distal bone, were considered to be minor amputations.^46^ A major amputation was performed in patients where the peripheral circulation could not be optimized (judgment of the referral angiologist) and would therefore not allow for adequate healing of a more distal amputation level and, in the setting of compromised soft tissue (remaining soft tissue would not allow to create a bearable more distal stump), or in case with widespread osteomyelitis and fulminant, life-threatening sepsis. Antibiotic therapy was directed by an in-house infectiologist based on the current IWGDF (International Working Group of the Diabetic Foot) guidelines.^30^ The administration route and the length of treatment were chosen according to (1) the severity of residual soft tissue infection, (2) residual osteomyelitis, and (3) in case of septic patients, presence of positive blood cultures to prevent development of endocarditis.

For each episode, defined as the occurrence of diabetic foot osteomyelitis at a particular anatomical location that demonstrated healed operative wounds and absence of clinical signs of reinfection within 12 months after the index amputation, we assessed the following variables: sex, body mass index, diabetes type and duration, cardiac artery disease (history of myocardial infarction, angina pectoris, congestive heart failure), nephropathy (glomerular filtration rate < 45 mL/min, patients requiring dialysis and/or history of kidney transplantation), polyneuropathy, and the history of smoking and alcohol abuse. Polyneuropathy was diagnosed using the 5.07 Semmes-Weinstein monofilament test and vibration test. If diagnosis of polyneuropathy was unclear, a referral to a neurologist was made. We also assessed laboratory results such as glycosylated hemoglobin on admission, inflammatory markers, and glomerular filtration rate. Furthermore, amputation levels and the type and duration of systemic antibiotic therapy based on the microbiological culture from the intraoperative bone probes were analyzed. Off-loading by casts or customized shoe orthosis alleviated any pressure areas to prevent further future foot lesions. Patients were seen on an outpatient basis every 1-2 weeks until complete wound healing was achieved. Afterwards, patients were seen regularly every 3 months.

### Outcomes

Our primary endpoint was the occurrence of ultimate DFO-related major amputation in regard to the initial anatomical localization of DFO. Phalanges and metatarsals were considered as forefoot, the distal row of the tarsus as midfoot, and the talus and calcaneus as hindfoot. The secondary objectives were different variables associated with an ultimate DFO-related major amputation.

### Statistical Analysis

We compared the differences between the localizations using a Student *t* test for mean values and the standard deviation (SD) for the continuous variables. For the comparison of categorical data, we used the Pearson chi-square test. The possible influence of different patient characteristics, demographic data, and clinical parameters on the incidence of major amputation were evaluated with a univariate and multivariate logistic regression analysis. We used IBM SPSS software (version 26; IBM, Armonk, NY) and Stata (version 13.1; StataCorp LP, College Station, TX) and considered *P* values <.05 (2-tailed) as statistically significant.

## Results

### General Results

We included 583 DFO episodes in 344 patients (78 females, 266 males; mean age 66.5 years). A total of 512 of 583 DFO episodes (87.8%) were located in the forefoot, 43 of 583 episodes (7.4%) in the midfoot, and 28 of 583 (4.8%) in the hindfoot (Table 1). The mean follow-up time was 3.6 years (SD: 3.52; range 0-18) after the last performed surgery. The average HbA_1c_ was 7.9 ± 1.6 (*P* = .915) and 8.0 (SD 1.8) (*P* = .915) in patients with and without MA, respectively. The mean body mass index did not differ significantly between the 2 groups (29.9 vs 30.3, *P* = .456). Other patients’ demographics are listed in Table 2. When comparing episodes treated with a major amputation to those with minor amputations, a bypass or percutaneous transluminal angiography was performed in 65.2% vs 48.4% of forefoot, 100% vs 38.2% of midfoot, or 50% vs 60% of hindfoot DFO. These differences were not significant. The mean interval between percutaneous transluminal angiography and surgery was 15 months (SD 29.5), 5.8 months (SD 12.8), and 9.2 months (SD 15.1) for fore-, mid-, and hindfoot DFOs, respectively. DFO in the setting of Charcot neuro-osteoarthropathy (CN) was significantly more prevalent in midfoot amputations (27/38, 71.1%) than in hindfoot amputations (6/19, 31.6%), direct major amputations (6/22, 27.3%), or forefoot amputations (44/504, 8.7%). In 27.3% of all direct major amputations, CN was present, whereas 1 of 23 (4.4%) forefoot, 1 of 4 (25.0%) midfoot, and 2 of 4 (50.0%) hindfoot DFO episodes were affected in secondary major amputations. Details of antibiotic therapy are given in Table 2. The most common bacterial species identified by intraoperative cultures from bone biopsies in patients with both minor and major amputations were *Staphylococcus aureus*, followed by coagulase-negative staphylococci and *Staphylococcus epidermidis* in patients with major amputations and *Enterococcus faecalis* and *Pseudomonas aeruginosa* in patients without major amputations (Table 3).

**Table 1.** Influence of Initial Amputation Location on Following DFO-Related Major Amputations.

Level of Initial Amputation	With MA	Without MA	Proportion MA of Amputation Level, %	Proportion of all DFO-Related MA, %
Forefoot				
Toes	8	262	3	15.1
Sesamoid	0	10	0	0
Ray	10	164	5.8	18.8
Total TM	5	45	10	9.4
Overall	23	481	4.6	43.3
Midfoot				
Lisfranc/Chopart	4	6	40	7.6
Internal resection	0	28	0	0
Overall	4	34	10.5	7.6
Hindfoot				
Partial calcanectomy	1	9	10	1.9
Syme/Pirogoff	1	3	25	1.9
Internal resection	2	3	40	3.8
Overall	4	15	21.1	7.6
Primary MA	22	—	100	41.5
Total	53	530	—	100

Abbreviations: DFO, diabetic foot osteomyelitis; IPFA, internal partial foot amputation; MA, major amputation; TM, transmetatarsal.

**Table 2.** Episode-Specific Characteristics Between Groups Treated Without and With Major Amputation.

Variable	Episodes Without MA	Episodes With MA	*P* Value
Age, mean (SD)	67 (11.6)	66 (11.6)	.707
Sex, n (%)			>.999
Female	115 (21.7)	11 (20.8)	
Male	415 (78.3)	42 (79.2)	
BMI, mean (SD)	29.9 (5.9)	30.3 (7.3)	.456
DM, n (%)			.273
Type I	68 (12.8)	9 (17.0)	
Type II	458 (86.4)	43 (81.1)	
Other	4 (0.8)	1 (1.9)	
Duration of DM, y, mean (SD)	19.1 (12.0)	21.4 (12.8)	.236
Peripheral neuropathy, n (%)	486 (91.7)	49 (92.5)	>.999
Renal pathology, n (%)	120 (22.6)	14 (26.4)	.499
Coronary artery disease, n (%)	211 (39.8)	30 (56.6)	.018^a^
Peripheral artery disease (PAD), n (%)	355 (67.0)	47 (88.7)	.001^a^
History of smoking, n (%)	304 (57.6)	40 (75.5)	.011^a^
Persistent smoking (% of smoker)	111 (36.2)	15 (37.5)	.863
Alcohol abuse, n (%)	123 (23.3)	19 (35.8)	.043^a^
Contralateral MA, n (%)	17 (3.2)	5 (9.4)	.023^a^
HbA_1c_, %, mean (SD)	8.0 (1.8)	7.9 (1.6)	.915
Ipsilateral CN, n (%)	73 (13.8)	10 (18.9)	.305
GFR (mL/min/1.73 m^2^), mean (SD)	64 (25)	61 (28)	.637
Preoperative antibiotic therapy, n (%)	373 (70.4)	44 (83.0)	.056
Duration of preoperative antibiotic therapy, d, mean (SD)	20 (31)	22 (30)	.991
Postoperative antibiotic therapy, n (%)	527 (99.4)	51 (96.2)	.068
Duration of postoperative antibiotic therapy, d, mean (SD)	32 (32)	29 (41)	.015^a^
Postoperative IV antibiotic therapy, n (%)	357 (69.3)	42 (79.2)	.156
Duration of postoperative IV antibiotic therapy, d, mean (SD)	11 (17)	9 (7)	.596
Mortality, n (%)	152 (28.7)	15 (28.3)	>.999
Interval amputation to death, mo, mean (SD)	55 (46.3)	38 (28.0)	.264
Total episodes	530	53	

Abbreviations: BMI, body mass index; CN, Charcot neuro-osteoarthropathy; DM, diabetes mellitus; GFR, glomerular filtration rate; IV, intravenous; MA, major amputation.

a Significant results.

**Table 3.** Occurence of Most Common Isolated Microorganisms in All DFO Episodes (n=583).

Isolated Organism	With MA, n (%)	Without MA, n (%)	*P* Value
No microorganism	49 (9.3)	6 (11.3)	ns
No culture	2 (0.4)	13 (24.5)	ns
No documentation	78 (14.7)	5 (9.4)	ns
*Enterobacter cloacae*	22 (4.2)	3 (5.7)	ns
*Escherichia coli*	12 (2.3)	4 (7.5)	**.025**
*Enterococcus faecalis*	37 (7.0)	7 (13.2)	ns
Coagulase-negative staphylococci	69 (13.0)	4 (7.5)	ns
*Staphylococcus epidermidis*	65 (12.3)	3 (5.7)	ns
*Staphylococcus epidermidis* (multidrug resistant)	26 (4.9)	2 (3.8)	ns
*Staphylococcus aureus*	133 (25.1)	8 (15.1)	ns
*Pseudomonas aeruginosa*	39 (7.4)	5 (9.4)	ns
*Stenotrophomonas maltophilia*	2 (0.4)	2 (3.8)	**.004**
*Enterococcus avium*	1 (0.2)	1 (1.9)	**.044**
*Peptostreptococcus magnus*	1 (0.2)	2 (3.8)	**.01**

Abbreviations: DFO, diabetic foot osteomyelitis; MA, major amputation; ns, nonsignificant.

### Major Amputations

All major amputations performed were transtibial amputations. In 22 of 583 (3.8%) cases, a new episode was directly treated with a primary major amputation. In 8 (1.4%), 5 (0.9%) and 9 (1.5%) of these 583 cases, osteomyelitis was located in the fore-, mid- and hindfoot, respectively. Thus, primary major amputations due to DFO were most often performed in the hindfoot (9/28, 32.1%), followed by the midfoot (5/43, 11.6%) and forefoot (8/512, 1.6%). Secondary major amputations (31/53, 58.5%) were most often required in the setting of hindfoot DFO (21.1%), followed by midfoot (10.5%) and forefoot (4.6%) DFO (Table 1). Overall, we performed a major amputation in 63 cases, of which 53 (84.1%) were due to DFO as the main indication. Among these (53/583), limb loss occurred in 6.1% (31/512) in the forefoot, in 20.9% (9/43) in the midfoot, and in 46.4% (13/28) in the hindfoot. In the remaining 10 of 63 cases (15.9%), indications for major amputation other than DFO were acute embolic occlusion (1/10), mechanical problems due to deranged biomechanics after minor amputations and CN (1/10), chronic ulceration after minor amputation (1/10), and critical ischemia leading to severe infected skin necrosis (7/10). Men (42/53, 79.3%) were affected more commonly by major amputations than females. Twenty of 53 (37.7%) transtibial amputations occurred after complete wound healing was achieved. Twenty-one of 53 (39.6%) experienced wound healing problems after transtibial amputation, with 12 of 21 (57.1%) requiring local revision surgery of the stump. Overall, only 1 of 53 of the transtibial amputation later required an above-knee amputation due to extensive damage to soft tissue, infected stump, and advanced PAD. The overall mortality rate after major amputation was 28.3% (15/53) after a mean time of 38 months (SD 28). No significant difference (*P* = .073) regarding time to death following MA between patients with (43 months, SD 24 months) and without (28 months, SD 31) critical ischemia (defined as Fontaine stages 3 and 4)^44^ was observed.

### Conversion to Secondary DFO-Related Major Amputations

In 31 of 583 (4.3%) cases, the primary minor amputation failed, leading to a conversion to a secondary DFO-related major amputation. Among this group, the prior DFO was localized in the forefoot in 23 of 512 (4.5%), the midfoot in 4 of 38 (10.5%), and the hindfoot in 4 of 19 cases (21.1%), respectively (Table 1). The mean time to conversion for all amputation levels was 9.3 months (SD 14.0). It was 42 months (SD 48.0) in the forefoot, 4 months (SD 1.0) in the midfoot, and 4.9 months (SD 4.7) in the hindfoot. Indications for conversion to secondary DFO-related MA are summarized in Table 4.

**Table 4.** Operative Indications for the Secondary Major Amputations (n=31).

Reason for DFO-Related MA	Number of Cases	Percentage
Progression of DFO (ascending infection)	2	6.5
Progression of DFO with severe PAD	6	19.3
Soft tissue damage due to deformity of foot	3	9.7
Soft tissue damage with severe PAD	5	16
Hindfoot DFO and sepsis	4	12.9
Hindfoot DFO and soft tissue damage	2	6.5
Chronic ulceration and severe PAD	2	6.5
Necrosis of amputation stump	7	22.6
Total	31	100

Abbreviations: DFO, diabetic foot osteomyelitis; MA, major amputation; PAD, peripheral artery disease.

#### Risk factors for DFO-related major amputations

In multivariate analysis, a hindfoot DFO location remained significantly associated with major amputations whereas no other factors (eg, PAD, coronary artery disease, smoking, CN, alcohol abuse, and prior contralateral major amputation) were identified as associated with major amputations (Tables 5 and 6).

**Table 5.** Uni- and Multivariate Analysis of All DFO-Related Major Amputations (n=53).

	Univariate		Multivariate	
	OR (*P* Value)	95% CI	OR (*P* Value)	95% CI
Midfoot	2.5 (ns)	0.8-7.5	2.7 (.084)	0.9-8.5
Hindfoot	**5.6 (.004)**	**1.7-18.1**	**5.4 (.006)**	**1.6-18.0**
Peripheral artery disease	**3.9 (.002)**	**1.6-9.2**	2.4 (.089)	0.9-6.5
Smoking	**2.3 (.013)**	**1.2-4.3**	2.1 (.090)	0.9-5.1
Alcohol abuse	**1.8 (.045)**	**1.0-3.3**	1.3 (0.522)	0.6-3.0
Renal disease	1.2 (ns)	0.6-2.3	–	–
Coronary artery disease	2.0 (.020)	**1.1-3.5**	1.0 (.954)	0.4-2.2
CN	1.5 (ns)	0.7-3.0	0.6 (.452)	0.2-2.2
BMI	1.0 (ns)	0.9-1.1	–	
HbA_1c_	1.0 (ns)	0.8-1.2	–	
GFR	1.0 (ns)	0.9-1.0	–	
Preoperative antibiotic therapy	2.1 (ns)	0.9-4.3	–	
Duration of preoperative antibiotic therapy	1.0 (ns)	0.9-1.0	–	
Duration of postoperative antibiotic therapy	1.0 (ns)	0.9-1.0	–	
Postoperative IV antibiotic therapy	1.7 (ns)	0.8-3.4	–	
Duration of postoperative IV antibiotic therapy	1.0 (ns)	0.9-1.0	–	
Prior contralateral minor amputation	1.5 (ns)	0.8-2.7	0.9 (.903)	0.4-2.1
Prior contralateral major amputation	**3.8 (.016)**	**1.3-11.1**	2.4 (.221)	0.6-9.8

Abbreviations: BMI, body mass index; CN, Charcot neuro-osteoarthropathy; DFO, diabetic foot osteomyelitis; GFR, glomerular infiltration rate; IV, intravenous; ns, not significant; OR, odds ratio.

**Table 6.** Uni- and Multivariate Analysis of Secondary DFO-Related Major Amputations (n=31).

	Univariate		Multivariate	
	OR (*P* Value)	95% CI	OR (*P* Value)	95% CI
Midfoot	2.5 (ns)	0.8-7.5	2.7 (.084)	0.9-8.5
Hindfoot	**5.6 (.004)**	1.7-18.1	**5.4 (.006)**	**1.6-18.0**
Peripheral artery disease	2.6 (.058)	1.0-6.8	2.4 (.089)	0.9-6.5
Smoking	**2.5 (.035)**	1.1-6.0	2.1 (.090)	0.9-5.1
Alcohol abuse	1.6 (ns)	0.7-3.4	1.3 (.522)	0.6-3.0
Renal disease	1.9 (ns)	0.9-4.0	–	
Coronary artery disease	1.4 (ns)	0.7-3.0	1.0 (.954)	0.4-2.2
CN	0.9 (ns)	0.3-2.7	0.6 (.452)	0.2-2.2
BMI	1.0 (ns)	0.9-1.1	–	
HbA_1c_	1.1 (ns)	0.9-1.6	–	
GFR	1.0 (ns)	0.9-1.0	–	
Preoperative antibiotic therapy	1.8 (ns)	0.7-4.4	–	
Duration of preoperative antibiotic therapy	1.0 (ns)	0.9-1.0	–	
Duration of postoperative antibiotic therapy	1.0 (ns)	0.9-1.0	–	
Postoperative IV antibiotic therapy	1.5 (ns)	0.6-3.6	–	
Duration of postoperative IV antibiotic therapy	1.0 (ns)	0.9-1.0	–	
Prior contralateral minor amputation	1.1 (ns)	0.5-2.4	1.0 (.903)	0.4-2.1
Prior contralateral major amputation	3.4 (.071)	0.9-12.8	2.4 (.221)	0.6-9.8

Abbreviations: BMI, body mass index; CN, Charcot neuro-osteoarthropathy; DFO, diabetic foot osteomyelitis; GFR, glomerular infiltration rate; IV, intravenous; ns, not significant; OR, odds ratio.

#### Influence of antibiotic therapy

Five variables of antibiotic therapy were tested for being additional risk factors for DFO-related major amputations: Preoperative antibiotic therapy, duration of preoperative antibiotic therapy, overall length of postoperative antibiotic therapy, intravenous postoperative antibiotic therapy, and length of intravenous postoperative antibiotic therapy. All variables were insignificant in the univariate analysis for both DFO-related major amputations and secondary DFO-related major amputations (Tables 5 and 6).

## Discussion

Although the diagnosis of DFO can be challenging, its ideal treatment remains controversial, even though several guidelines exist.^1,5,30^ According to our retrospective study, hindfoot DFO carries a fivefold risk for DFO-related major amputation compared with forefoot DFO.

Faglia et al^14^ found a significant association between hindfoot location of DFO and major amputation in a series of 350 patients. The data of our study confirmed this result in a 1.7-fold larger episode sample group. There are several differences between both studies that are important to note. Contrary to Faglia et al, we included patients with CN to test CN as an additional potential risk factor for major amputation. Further, we introduced preoperative antibiotic therapy as well as duration and administration route of postoperative antibiotic therapy in the statistical analysis. Our statistical model differed as we tested the 3 anatomical regions against each other whereas Faglia et al did not introduce the midfoot area as a separate variable in their analysis. Finally, we performed separate analyses for overall and secondary major amputations.

Overall, the major amputation rates for hind- and midfoot DFOs were 46% and 21%, in contrast to only 6% for forefoot DFO. Similarly to Faglia et al’s findings, Cevera et al^11^ postulated that limb salvage is 2-3 times less likely with heel ulcers. Additionally, further options for operative resection in hindfoot DFO are limited, and therefore major amputation may be the only remaining treatment option.^32^ From a biomechanical standpoint, 2 aspects contribute to those limited options. Weightbearing must be absorbed by a single bony surface that can be limited by loss of bone stock or deformity (eg, hindfoot CN).^42^ Further, the heel pad can be compromised by PAD, soft tissue infection, or loss.^40^ Both aspects can be compromised in the feet of patients with diabetes. One study found 50% below-knee amputation rate after total calcanectomy due to calcaneal osteomyelitis. However, patients without diabetes were also included in this study.^59^ After performing well initially, in the long term, higher mortality and reamputation rates have been reported in partial and total calcanectomies when compared with transtibial amputations.^9^ Specifically, 16.7% of partial calcanectomies resulted in a later transtibial amputation.^52^ This might raise the question if a direct major amputation is justified in the setting of hind- and midfoot DFO in selected patients, reducing the need for repeated operative interventions, hospitalization length, in-hospital postoperative complications, and convalescence.^21,55^ In contrast, others favor an attempt to salvage the limb even in spite of higher failure rates because major amputation is known to be associated with higher energy expenditure and in some cases reduced ambulatory status.^4,33,53^

We observed more conversions from Lisfranc and Chopart amputations to major amputations than Syme or Spitzy-Pirogoff amputations. The explanation is likely biomechanical. In contrast to Syme or Pirogoff amputations, both Lisfranc and Chopart amputations result in an overpull of the Achilles tendon and are therefore prone to ulcer development if the surgeon foregoes an Achilles tendon–lengthening procedure.^40^ Additionally, in Lisfranc amputations, lack of preservation of the base of the fifth metatarsal or proximal reinsertion of the Peroneus brevis tendon can result in equinovarus deformities.^27^ Chopart amputations need additional reinsertion of the tibialis anterior and the peroneal tendons to prevent equinovarus.^3^ However, in diabetic foot infections, some surgeons refrain from performing tendon balancing to prevent further infection spread in healthy bone and rely on the prosthetic device to prevent ulcer development.^15,20^ Although advantages of Chopart amputations include a functional stump, limb length preservation and the ability of autonomous walking, challenges with fitting of the prosthesis, difficulty balancing biomechanical forces, as well as wound healing problems are well described.^8,15,26^ In contrast, patients with diabetes with Syme amputation benefit from the ability to directly bear weight, normal lever during the push-off phase, and improved proprioception and stability during gait.^43^ In the long term, patients achieved improved levels of functional independence and required less rehabilitation than patients who underwent a transtibial amputation.^17^ In our study, 25% of Syme amputations later required a major amputation, which is in the range described in the previous literature (14.7%-38.5%).^7,17,19^

Although most of our major amputations were due to DFO located in the forefoot, this occurred in 6% of forefoot DFO cases. Moon et al observed that 4.7% of forefoot ulcers in the setting of diabetes mellitus underwent later major amputation. Risk factors such as male gender, elevated HbA_1c_, increased magnesium and platelet levels were associated in univariate analysis with major amputation.^35^ In contrast, Yeager et al showed a higher percentage (18.5%) of patients with forefoot foot lesions requiring later major amputation in the follow-up period. They noted that the most important factor for major amputation after failed forefoot minor amputation was an unsuccessful revascularization.^63^ We attribute the far lower percentage in our study to improvement in vascularization techniques, as well as standardized aftercare with off-loading orthopaedic insoles or shoes and frequent follow-up appointments every 3 months over the clinical course, which were continued even in absence of skin lesions.

In contrast to the above listed findings, some studies have not shown any correlation between ulcer site or location of DFO on amputation rate.^23,39^ However, one study only analyzed the overall amputation rate and did not differentiate between minor and major amputations.^39^

Regarding diabetic feet, CN is of particular interest as it has been reported to be a risk factor for major amputation.^13,36^ CN was most prevalent in midfoot DFO, capable of causing major destruction and foot deformation, resulting in pressure areas that facilitate skin lesions and wound healing problems. In multivariate analysis, CN was not identified as an independent risk factor for primary or secondary major amputation; however, the prevalence of CN in our cohort was less than 20%. We also attribute some of our success with this population to rigorous CN aftercare protocol with appointments every 3-6 months even in the setting of an Eichenholtz stage 3 CN and treatment with orthopaedic shoes made by experienced orthopaedic shoemakers. Nevertheless, presence of CN must be considered as it can affect the contralateral foot, which may later require contralateral amputation (16.9% overall, 2.3% major amputation) as well.^58^

We were unable to detect an influence of preoperative antibiotic therapy and its duration, the overall duration of postoperative antibiotic therapy, and intravenous postoperative antibiotic therapy and its duration on major amputations. Similar to our findings, both administration route and duration of antibiotics have been investigated as potential risk factors for therapy failure in diabetic foot infections in general, failing to demonstrate any influence.^22^ Duration and administration route of antibiotic therapy in DFO remain a much-debated topic with uncertainty regarding the need of intravenous administration and optimal length of antibiotic therapy even in the most recent IWGDF guidelines.^30^

Other patient factors that have been associated with DFO-related major amputation include end-stage renal disease, hypertension, coronary artery disease, PAD, and cerebrovascular disease as well as the duration of diabetes, the occurrence of multiple ulcers, and deep ulcers reaching the bone.^14,16,23,31,33,49,61^ Further, pulmonary and bleeding disorders, increased HbA_1c_, smoking, elevated inflammatory markers such as C-reactive protein, erythrocyte sedimentation rate, and white blood cells have been described (summarized in Table 7).^2,31,33,34,64^ Our data did not identify any additional independent risk factors in addition to the hindfoot location. It is a general problem that the diabetic foot population is heterogenous so that generalization of particular findings on the diabetic foot is difficult, and most studies have not had sufficient size to perform multivariate analyses.^31,47,57^

**Table 7.** Proportion of DFO Later Requiring a Major Amputation (MA) and the Associated Risk Factors Compared With the Results of Our Study.

Location	Proportion of MA in Literature (%)	Risk Factors for MA	Proportion of MA in Our Study (%)
Forefoot	Overall Moon et al^35^: 4.7% Yeager et al^63^:18.5% Transmetatarsal Pinzur et al^41^:14.1% Younger et al^64^: 35.3%	Male gender, elevated HbA1c, increased magnesium and platelet levels Unsuccessful revascularization Low albumin levels, low lymphocyte count, low ischemic index HbA1c	Overall: 4.6% Ray: 5.0% TM: 10.0%
Midfoot	Overall: Moon et al^36^: 8.9% Chopart-amputation: Brodell et al^8^: 44% Faglia et al^15^: 28%	Charcot foot, cardiac disorder, deep ulcers PAD No significant RF	Overall: 10.5%
Hindfoot	Overall: Moon et al^34^: 11.1% Calcanectomy: Waibel et al^59^: 50% Syme-amputation Finkler et al^17^: 12.1% Frykberg et al^19^: 38.5%	Pulmonary disorder, ESR, TIBC levels PAD, dialysis, smoking, alcohol abuse No significant RF No significant RF	Overall: 21.1% Partial Calcanectomy: 10% Syme/Pirogoff: 25%

Abbreviations: DFO, diabetic foot osteomyelitis; ESR, erythrocyte sedimentation rate; MA, major amputation; PAD, peripheral artery disease; RF, risk factors; TIBC, total iron-binding capacity; TM, transmetatarsal.

Mortality rates of 29.4% to 52% have been reported after transtibial amputations.^13,61^ In our study, we noticed a mortality rate of 28.3% after major amputation, which may be related to advanced treatment modalities of patients’ comorbidities.

This study has limitations. First, only patients with operative treatment of DFO were included in the evaluation. Therefore, a conclusion on the efficacy of antibiotic treatment alone cannot be drawn with the here presented results. Second, our study follows a retrospective design, and therefore transferability to clinical practice might be partially limited. Third, we lacked data on patients’ nutritional status. Prior studies have demonstrated that lower albumin levels—as a marker for nutritional status—are associated with treatment failure or limb loss.^12,41,43,62^ Additionally, DFO may occur simultaneously to gangrene, soft tissue infection, or sepsis. Furthermore, because of the small sample size in some subgroups, the power to detect other associations was limited. Lastly, in a retrospective study design, we cannot control for the overall therapeutic and preventive compliance of the patient, which is a general problem in the entire field of science investigating outcomes in patients with long-standing diabetes mellitus.

## Conclusion

The frequency of limb loss in DFO increases the more proximal the initial DFO lesion occurs in the foot, with almost half of patients losing their limbs suffering from hindfoot DFO. Only hindfoot DFO was found to be an independent risk factor for DFO-related major amputation both initially and after initial partial foot amputation care.

## Supplemental Material

sj-pdf-1-fai-10.1177_10711007221088552 – Supplemental material for Foot Osteomyelitis Location and Rates of Primary or Secondary Major Amputations in Patients With Diabetes Click here for additional data file. Supplemental material, sj-pdf-1-fai-10.1177_10711007221088552 for Foot Osteomyelitis Location and Rates of Primary or Secondary Major Amputations in Patients With Diabetes by Elin Winkler, Madlaina Schöni, Nicola Krähenbühl, Ilker Uçkay and Felix W. A. Waibel in Foot & Ankle International
